# En las entrañas del SARS-CoV-2: liderazgo científico del Instituto Nacional de Salud

**Published:** 2021-06-15

**Authors:** Luis Alberto Gómez-Grosso, Marcela Mercado, Martha Lucía Ospina

**Affiliations:** 1 Grupo de Fisiología Molecular, Instituto Nacional de Salud, Bogotá, D.C., Colombia Instituto Nacional de Salud BogotáD.C. Colombia; 2 Directora de Investigación en Salud Pública, Instituto Nacional de Salud, Bogotá, D.C., Colombia Instituto Nacional de Salud BogotáD.C. Colombia; 3 Directora General, Instituto Nacional de Salud, Bogotá, D.C., Colombia Instituto Nacional de Salud BogotáD.C. Colombia

La pandemia de Covid-19 causada por el coronavirus SARS-CoV-2 sigue su curso y se ha extendido a más de 200 países. Hasta el 21 de junio de 2021 -fecha en la que se terminó de escribir este editorial- se registraban más de 180 millones de casos confirmados y cerca de 4 millones de muertes en todo el mundo, en tanto que, en Colombia, eran cerca de 4 millones los casos confirmados y más de 100.000 las muertes, lo que subraya la gravedad de la enfermedad (1,2).

**Figura 1:**
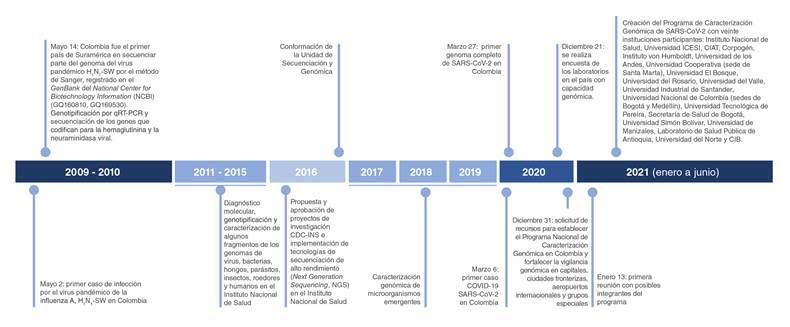
Línea de tiempo: pruebas moleculares en la atención de emergencias en salud pública en el Instituto Nacional de Salud

Desde el inicio de la emergencia sanitaria por la Covid-19, y a lo largo de estos 15 meses, el Instituto Nacional de Salud ha abordado varias áreas de investigación y estudios relacionados con el análisis de la pandemia por SARS-CoV-2 que, aunque constituyen campos diferentes, están ambos asociados con la comprensión del virus y su impacto en la salud pública. Dado el gran potencial del virus de transmitirse de persona a persona, incluso de portadores asintomáticos, y de personas a animales (3-6), antes del primer pico, el enfoque a nivel local y global fue el de desarrollar y estandarizar pruebas de diagnóstico rápidas y precisas, fundamentales para adoptar acciones inmediatas de prevención, así como para el tratamiento oportuno y el control de los brotes. Las pruebas moleculares se convirtieron en el gran reto y el Instituto Nacional de Salud, con base en su trayectoria y su experiencia en biología molecular y en la atención de emergencias en salud pública -por el H_1_N_1_, el zika y el chikunguña, entre otras-, fue el primero en el país en desarrollar la PCR cuantitativa en tiempo real (qRT-PCR), método de referencia para detectar el nuevo coronavirus (figura 1).

Desde un inicio, y con base en la experiencia adquirida y las lecciones aprendidas, se reconoció la utilidad de la biología molecular para detectar y caracterizar de manera oportuna y rápida los virus circulantes, así como los principales factores determinantes, pues dicha información, compartida nacional e internacionalmente, es necesaria para el control oportuno de una pandemia (7). El tiempo de respuesta y el volumen de casos requirieron de un esfuerzo integral sin precedentes por parte del Instituto. Para dar respuesta a la emergencia sanitaria, se organizaron equipos de trabajo funcionales, se generó la capacidad de trabajo en red y se plantearon las siguientes cinco líneas estratégicas de acción:

1. vigilancia y respuesta en salud pública a nivel nacional;
2. generación de una capacidad diagnóstica de calidad y con precisión a nivel nacional;
3. desarrollo y uso de modelos matemáticos complejos para aproximarse a la realidad de la transmisión y la dinámica de la pandemia;
4. investigación para responder preguntas en el menor tiempo posible, y
5. traducción de la información y del nuevo conocimiento para entregarlos al gobierno, a la comunidad científica y a la población general.

En pocos días, el Instituto Nacional de Salud implementó las pruebas moleculares para detectar el genoma del SARS-CoV-2. Dada la cantidad de muestras, se requerían instrumentos especializados y costosos, es decir, las pruebas debían llevarse a cabo en laboratorios dotados de los equipos y la experiencia necesarios. Ello incluyó el desarrollo de un proceso específico para la toma y transferencia de las muestras desde los puntos de recolección a las instalaciones de prueba, y los procesos para la extracción y purificación del ARN viral, la síntesis de cNDA y los miles de reacciones de amplificación del ácido nucleico específicas para este virus, con el fin de cubrir la demanda nacional de pruebas de detección de las personas infectadas lo más rápidamente posible. Además, el Instituto Nacional de Salud gestionó recursos y donaciones para fortalecer los laboratorios de salud pública y otros 160 laboratorios, con el fin de enfrentar el reto de procesar más de 80.000 pruebas de diagnóstico al día. También, desarrolló modelos matemáticos que permitieron simular diferentes escenarios de la pandemia en las entidades territoriales, lo cual requirió una intensa capacitación y acompañamiento por parte del Instituto.

En esta fase del estudio de la pandemia, se desarrollaron la capacidad diagnóstica, la dinámica de la apropiación social del conocimiento y las capacidades técnicas por parte de laboratorios públicos y privados, y se establecieron las directrices para la evaluación directa e indirecta del desempeño de los laboratorios departamentales de salud pública y para la validación de las pruebas de detección de antígenos capaces de detectar la presencia de proteínas virales específicas. Asimismo, se creó el repositorio nacional de resultados de las pruebas de Covid-19 (Sismuestra), y se implementaron otras herramientas de operatividad interinstitucional para la vigilancia y el diagnóstico, como la plataforma CoronApp Colombia, que permitió la notificación de casos en tiempo real por parte de los laboratorios (8). Este liderazgo permitió proyectar y pronosticar las curvas de crecimiento y la dinámica de la pandemia. No obstante, se mantenía la incertidumbre sobre cuántos eran los casos reales en la población colombiana y a nivel global, y si el virus generaba algún tipo de inmunidad.

Para responder a estos interrogantes, el Instituto Nacional de Salud desarrolló en tiempo récord e implementó diferentes tipos de pruebas de diagnóstico rápido, incluidas las serológicas que detectan anticuerpos humanos contra el SARS-CoV-2, lo cual permitió determinar la seroprevalencia en Colombia y marcó una segunda fase de estudios científicos de la pandemia. Los datos obtenidos en diez ciudades de Colombia estaban respaldados por muestreos confiables realizados con el Departamento Administrativo Nacional de Estadística (DANE), los cuales reflejaban la magnitud de la infección tras el final del primer pico, y permitieron ajustar los modelos de infección y las estrategias de rastreo en cada ciudad (9).

Las pruebas moleculares y la detección de los anticuerpos se emplean para detectar a los individuos infectados e infecciosos. Sin embargo, dado que los anticuerpos solo son detectables en etapas posteriores a la infección, cuando ya se han cerrado las oportunidades para tratar y limitar la transmisión de la enfermedad, y que los virus frecuentemente cambian y modifican su genoma, especialmente bajo presión selectiva, la prueba de RT- PCR y el análisis de anticuerpos no permiten monitorear de manera directa las variaciones en el genoma de los virus, ni conocer de forma precisa su circulación geográfica y temporal, como tampoco, saber con exactitud cuándo y dónde pueden surgir nuevas variantes de los virus con mayor o menor capacidad de transmisión y patogenicidad (7). Ello explica que, a comienzos del 2021, cuando se pensó que el mundo comenzaba a superar la pandemia por el SARS-CoV-2 y en algunos países ya se había iniciado la inmunización, se entró en una fase de aparición de variantes con mayor capacidad de transmisión y una menor habilidad de los anticuerpos para neutralizar su acción.

Varios países, incluido Colombia, secuenciaron y depositaron miles de genomas del SARS-CoV-2 en el servicio de intercambio de información genética de secuencias de genomas organizado por la *Global Initiative on Sharing All Influenza Data* (GISAID; NextStrain, https://nextstrain.org) y mediante la aplicación CoV-GLUE (http://cov-glue.cvr.gla.ac.uk) (10,11). El análisis de sus secuencias reveló la existencia de muchas variantes (lo que sugiere que está en curso la adaptación del coronavirus a su nuevo huésped humano) (12,13), tales como las de Reino Unido, Suráfrica y Brasil, que se están extendiendo muy rápidamente y pueden afectar el rendimiento de las pruebas de diagnóstico y la reacción inmunológica (14). Las variaciones en el genoma viral también pueden crear desajustes de los anticuerpos en el sitio de unión (epítopos) del virus y afectar la capacidad de la interacción del complejo antígeno-anticuerpo, tanto para el diagnóstico como para la reacción inmunológica protectora.

Dada la naturaleza bioquímica del SARS-CoV-2, esta situación no era tan inesperada y no nos tomó por sorpresa. Durante las más recientes epidemias causadas por virus, la secuenciación mediante tecnologías de alto rendimiento, también conocida como secuenciación de próxima generación (*Next Generation Sequencing*, NGS), ya hacía parte de nuestra rutina de investigación y ello nos permitió la caracterización genómica, la vigilancia en tiempo real de la dispersión viral y la identificación de nuevas variantes; además, permitió explicar algunos mecanismos patogénicos y algunas dinámicas evolutivas del virus. Esta aproximación desde la investigación básica y aplicada en salud pública ha tenido un efecto positivo en el desarrollo científico del país. Desde el 2014, el Grupo de Genómica del Instituto Nacional de Salud lidera proyectos de investigación que han aportado a la caracterización genómica y al conocimiento científico de la dinámica de virus patógenos, principalmente de arbovirus como los de dengue (DENV), chikungunya (CHIKV) y zika (ZIKV) (15), de poxvirus emergentes en Colombia, de los causantes de hepatitis virales y, más recientemente, del virus SARS-CoV-2, todos ellos de gran impacto en la salud pública y en la economía del país.

Con una visión prospectiva y como parte de sus acciones estratégicas, desde el inicio de la pandemia, el Instituto Nacional de Salud, en articulación con las directrices ya establecidas, planeó la secuenciación de todo el genoma del virus, cuyo contenido de es de 30.000 nucleótidos aproximadamente. Por lo tanto, como parte de la respuesta a los retos de la pandemia y para avanzar en el conocimiento de la estructura y fisiología molecular del SARS-CoV-2 circulante en Colombia, el Instituto adecuó el laboratorio de bioseguridad de nivel 3 (BSL-3) para el cultivo del virus, formalizó el grupo de investigación en genómica y creó la Red Nacional de Genómica del SARS-Cov-2. Esta estrategia respondió a la premisa de que la secuenciación de todo el genoma del virus es una herramienta poderosa que permite conocer el orden y el contenido de nucleótidos de los virus y, por lo tanto, identifica de manera inequívoca las variantes de este nuevo coronavirus, es decir, la genómica del SARS-CoV-2. Esto allana el camino para generar alertas tempranas frente a la dispersión de variantes emergentes, contribuye a fortalecer la vigilancia epidemiológica, facilita el desarrollo y el refinamiento de pruebas moleculares y serológicas para el diagnóstico, el estudio de la reacción inmunológica y el diseño de vacunas. Además, contribuye al estudio de la dinámica de la introducción y circulación del virus, su relación con el aumento o disminución de casos y sus características clínicas y, eventualmente, al diseño racional de medicamentos y de productos biotecnológicos contra el virus.

Por la naturaleza de esta pandemia, fue necesario proponer un programa nacional de caracterización genómica en Colombia, con el objetivo de contar con una red capaz de secuenciar de manera sistemática y permanente los genomas de SARS-CoV-2 representativos de todo el país, por lo menos, durante 18 meses. Esto permitiría determinar el patrón geográfico de los linajes predominantes, ya que la ejecución de los cinco proyectos aprobados por el Ministerio de Ciencia Tecnología e Innovación para la secuenciación del genoma del SARS-CoV-2 está proyectada a mediano plazo, con un número de secuencias limitado y un alcance específico en el marco de cada uno de ellos, concebidos, además, de manera independiente, por lo cual sus objetivos no contemplan una estrategia global de caracterización genómica para todo el país.

El 29 de marzo de 2020, el uso de la tecnología NGS nos permitió obtener la secuencia del genoma completo (16) en el caso de la primera infección reportada en el país, en una mujer que había estado en España, lo que confirmó que había sido causada por el SARS-CoV-2 cuyo genoma estaba estrechamente relacionado con la variante circulante en Wuhan. La vigilancia genómica se convirtió en un reto para el equipo de secuenciación y genómica del Instituto Nacional de Salud que, a la fecha, ha reportado más de 571 genomas completos del virus y generó el mapa de distribución de los 44 linajes circulantes en nuestro país (http://cov-lineages.org/lineages.html) (17). Es importante destacar que los investigadores del Instituto también han descubierto cambios en el genoma en los sitios objetivo mediante varias pruebas de qRT-PCR para el SARS-CoV-2 (23 genomas de la variante P1) (17-19). Estas variantes se generan, en parte, porque las desaminasas ADAR y APOBEC, que forman parte de las reacciones inmunológicas innatas del huésped humano a la infección, pueden editar el ARN del SARS-CoV-2. Los cambios de adenosina a inosina y de citosina a uracilo, respectivamente, generan nuevas variantes del virus (20), lo cual requiere un seguimiento y monitoreo continuos.

El Instituto Nacional de Salud buscó aliados académicos que tuvieran experiencia científica demostrada en la secuenciación de microorganismos y la capacidad técnica y humana para responder como país a esta fase de desarrollo de conocimiento e intervención de la pandemia, en la que es prioritario estar atentos a nuevas variantes y a su comportamiento en la población; más aún cuando todas las esperanzas están puestas en la vacunación como medida indispensable para alcanzar la inmunidad de rebaño y ganarle la batalla al SARS-CoV-2. En noviembre del 2020, el Instituto convocó a los laboratorios del país con capacidad de hacer secuenciación y análisis genómicos, procedimientos que claramente superan los análisis rutinarios de un laboratorio común, para establecer el Programa Nacional de Caracterización Genómica del SARS-CoV-2, proyecto que se convirtió en el gran reto de esta fase de análisis de la pandemia. La secuenciación sistemática y el análisis de los genomas del virus de todo el país nos están permitiendo conocer en tiempo real la circulación de los linajes de SARS-CoV-2 e identificar oportunamente aquellas variantes de preocupación, como las ya mencionadas, para así poder tomar decisiones tempranas que mejoren los procesos de vigilancia en salud pública y generen datos de acceso público que aporten al conocimiento de la dinámica del virus.

La investigación biomédica sobre la Covid-19 es prioritaria y es necesario compartir la información científica sobre el SARS-CoV-2 causante de la enfermedad (21). El liderazgo del Instituto en la respuesta a la pandemia de Covid-19 valida su existencia y revela la importancia de los institutos nacionales dedicados a la investigación científica. Sin duda, uno de los retos es continuar fortaleciendo el Instituto Nacional de Salud para incrementar su capacidad científica y tecnológica. Esto implica aumentar el talento humano con altos niveles de formación, fortalecer la tecnología para el procesamiento de información a gran escala (*big data*), e incrementar los procesos que requieren herramientas de bioinformática e inteligencia artificial. También, será un reto importante contribuir a diseñar un plan nacional para el desarrollo y producción de vacunas con tecnologías de punta, que dé respuestas a futuras pandemias por virus emergentes y reemergentes.
